# Quality Improvement Partnership to Improve Human Papillomavirus Vaccine Rates: Start at Age 9 Campaign

**DOI:** 10.1097/pq9.0000000000000927

**Published:** 2026-09-09

**Authors:** Lauren E. Ng, Jessica P. Fenton, Linda Mermelstein, Lori Ellis, Randi Trope

**Affiliations:** From the * Department of Pediatrics, Renaissance School of Medicine at Stony Brook University, Stony Brook, N.Y.; † Department of Family, Population, and Preventive Medicine, Renaissance School of Medicine at Stony Brook University, Stony Brook, N.Y.; ‡ Department of Community Outreach and Engagement, Stony Brook Cancer Center, Stony Brook University Hospital, Stony Brook, N.Y.

## Abstract

**Introduction:**

Human papillomavirus (HPV) infections and HPV-related cancers are preventable with vaccination. Initiating the HPV vaccine series at ages 9–10 years is associated with improved vaccination uptake and series completion. This quality improvement (QI) initiative aimed to increase first-dose HPV vaccination rates among 9- to 10-year-olds through the implementation of evidence-based interventions.

**Methods:**

Data were collected from electronic health records (EHRs) of established patients aged 9–10 years with at least 1 documented well-child visit annually from 2021 to 2024. Baseline data were obtained from January 2021 through May 2022. Interventions included provider education, adoption of a system-wide “Start at 9” policy, patient-facing educational materials, and EHR modifications to integrate HPV vaccine orders for patients aged 9 years and older. Monthly vaccination trends were evaluated using a P-chart.

**Results:**

A total of 9315 patients aged 9–10 years were included. The percentage of eligible patients receiving their first HPV vaccine increased from a baseline of 2% to 14% following the adoption of the “Start at 9” policy in conjunction with targeted educational interventions and EHR integration. A P-chart demonstrating special cause variation aligned with 2 QI interventions, indicating a sustained process shift. Tetanus, diphtheria, and acellular pertussis (Tdap) vaccination rates, used as balancing measures, remained stable throughout the study period.

**Conclusions:**

A coordinated, systems-level QI approach was associated with significant gains in early HPV vaccine initiation, supporting initiation of vaccination at age 9 years as an effective strategy to promote timely vaccination and strengthen preventive health outcomes.

## INTRODUCTION

Human papillomavirus (HPV) infections result in a substantial disease burden globally, causing cervical cancer, anogenital and oropharyngeal malignancies, and anogenital warts in both men and women.^1^ Recent studies demonstrate the benefits of early HPV vaccination in reducing the risk of HPV-related cancers. The introduction of HPV vaccination in England demonstrated an 87% reduction in cervical cancer and the incidence of grade 3 cervical intraepithelial neoplasia in young women, particularly among individuals offered the vaccine at age 12–13 years, as opposed to later HPV vaccination.^2^ A Swedish study showed an 88% reduction in the incidence of invasive cervical cancer among girls who were vaccinated before age 17 compared with girls who were not vaccinated.^3^ A 2021 literature review revealed a substantial decrease in vaccine-type oral or oropharyngeal HPV infections in study participants immunized with the HPV vaccine across study designs and heterogeneous populations.^4^ Although there are limited studies on the effectiveness of the HPV vaccine in reducing oral and pharyngeal cancers in men, a 2022 review of the literature made a strong case for pangender HPV vaccination in combating oral and pharyngeal cancer in men.^5^

Vaccination for the prevention of HPV infection has been available in the United States since 2006. HPV vaccine is most effective when given before any exposure to HPV; consequently, routine vaccination is offered to boys and girls at ages 11–12 years along with the tetanus, diphtheria, and acellular pertussis (Tdap) and meningitis vaccines. Initiation of the HPV vaccine series at ages 9–10 years can improve vaccination use and increase the number of adolescents who complete their series on time.^6^ Starting HPV vaccination between 9 and 10 years of age also contributes to higher adolescent vaccine coverage than initiation at later ages.^7^ The recommendation to start HPV vaccination at age 9 years has been supported by the American Academy of Pediatrics and the American Cancer Society.^8,9^ Despite these recommendations, HPV vaccination completion rates among 13-year-old adolescents in Suffolk County are 21.5%, which is lower than the New York State HPV vaccine series completion rate (25.7%).^10^ Low HPV vaccination rates among children and adolescents can be due to parental vaccine hesitancy, lack of a strong provider recommendation, and lack of resources to educate parents and patients about vaccines to overcome myths and misinformation.^11^

Quality improvement (QI) and learning collaboratives to improve HPV vaccination show promise by aligning activities with clinical workflows.^12^ Evidence-based interventions to increase HPV immunization include patient education, outreach for overdue well visits, physician/provider education, physician/provider electronic health record (EHR) support, and improved messaging to parents, such as bundling vaccines.^13^ Adaptations of evidence-based interventions should support the resources and individual capability of the healthcare organization or physician practice, with careful examination of the clinical workflows and feasible implementation of interventions.^14^

This QI project aims to increase the percentage of eligible patients who receive their first HPV vaccine dose by 10% and determine whether evidence-based interventions lead to sustainable improvement from July 1, 2022, to June 30, 2024. This project was implemented at 6 pediatric primary care clinical practice sites within a Northeastern academic medical center in New York State.

## METHODS

This quality initiative was conducted in partnership with the American Cancer Society, the Northeastern academic medical center in New York State, and the academic medical center’s Cancer Center Community Outreach and Engagement office. Data were derived from de-identified, retrospective extracts from the EHR system across the 6 pediatric primary care sites. Eligible patients included those aged 9 or 10 years with at least 1 well-child visit. Baseline data for eligible patients were obtained from January 1, 2021, through June 30, 2022. The study period was conducted between July 1, 2022, and June 30, 2024. HPV vaccination rates were calculated as the number of vaccinated patients divided by the number of eligible patients. Because the study included 6 different ambulatory sites, a patient who received care at more than 1 site could be counted as an eligible patient at multiple sites. Therefore, deduplicated HPV vaccination rates were calculated by identifying and counting each unique patient only once, regardless of the number of ambulatory sites at which the patient received care. Data were obtained and stratified by sex, race/ethnicity, and insurance type. These demographic variables were extracted from structured EHR fields and summarized using descriptive statistics. Additionally, first-dose Tdap vaccine rates were collected for patients in these age groups.

### Ethical Considerations

The project was undertaken as a QI project and as such did not constitute human subjects’ research.

#### Interventions

**Fig. 1.**
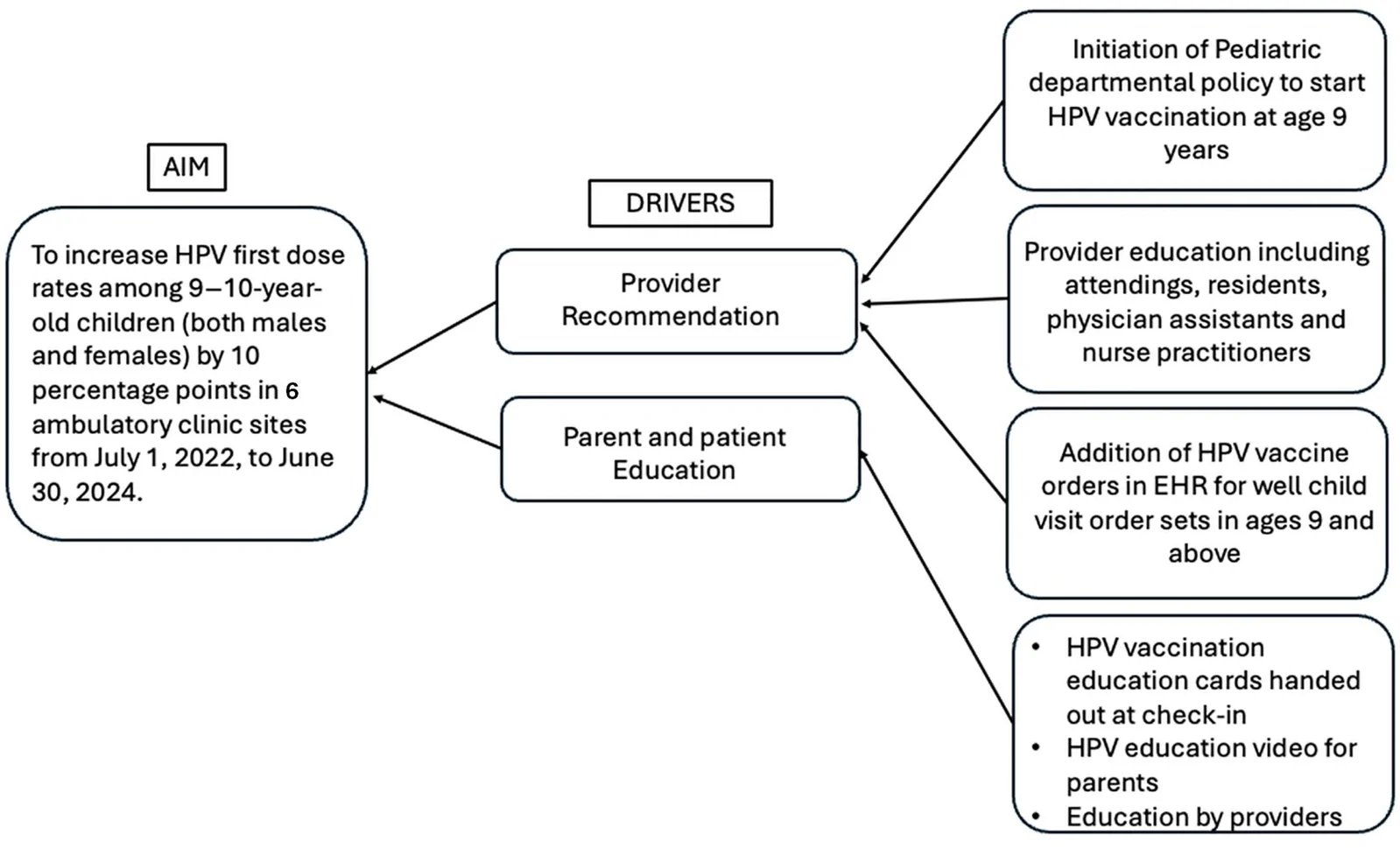
Key driver diagram.

Figure 1 presents the aim of the project and key driver diagram, illustrating the organizational framework, key drivers, and improvement initiatives.

The first improvement initiative implemented in July of 2022 included pediatric resident participation to conduct HPV vaccine education sessions at all sites for both providers and staff. These education sessions were conducted both virtually and in person through PowerPoint presentations. These sessions discussed starting HPV vaccination at age 9 years and included HPV vaccination rates specific to each office site.

The second improvement initiative implemented in January 2023 was a policy change. The academic medical center administration embraced the “Start at 9” campaign proposed by the New York State HPV Coalition.^15^ With the institutional policy change, HPV vaccine education regarding starting HPV vaccination at age 9 years continued for all healthcare team members, including physicians, residents, physician assistants, nurse practitioners, licensed practical nurses, registered nurses, and medical assistants. The education included a listing of the existing evidence for the benefits of starting HPV vaccination at age 9 years for cancer prevention, information about the institutional policy change, and information about the QI project and its objectives. This was conducted through both in-person and video-recorded PowerPoint presentations with continued presentation of updated HPV vaccine rates specific to each site.

The third improvement initiative began in April 2023 and involved patient education in partnership with the Cancer Prevention in Action (CPiA) team, a New York State grant program with Health Research Inc. that promotes HPV vaccination for cancer prevention through education and policy for HPV vaccination education at practices and health organizations. This collaboration was introduced by the Cancer Center Community Outreach and Engagement office at the medical center. At the time, there were no patient education materials specifically focused on initiating HPV vaccination at age 9 years. The CPiA team created an HPV vaccine information card geared toward parents and patients. It included information about HPV and the HPV vaccine and indicated that the vaccine series can be started at age 9 years. The card also included a QR code linking to a CPiA 3-minute educational video for parents. The HPV vaccine information cards were distributed to all patients aged 9 through 12 years at check-in for their well-child visits.

The fourth improvement initiative, implemented in October 2023, involved updating the EHR to include HPV vaccine orders and charges for all well-child visit order sets for ages 9 years and older.

#### Analysis

To evaluate the impact of the QI initiative, the primary outcome measure was the annual proportion of 9- and 10-year-old patients who received the first dose of the HPV vaccine. Balancing and process measures were also considered. The process measures corresponded to each improvement initiative and were assumed to have been implemented with full adherence. The proportion of patients in the same age group who received the Tdap vaccine was used as the balancing measure. This served to monitor whether improvements in HPV vaccination inadvertently impacted the delivery of other age-appropriate immunizations or if there was a general increase in vaccination not specific to HPV.

**Fig. 2.**
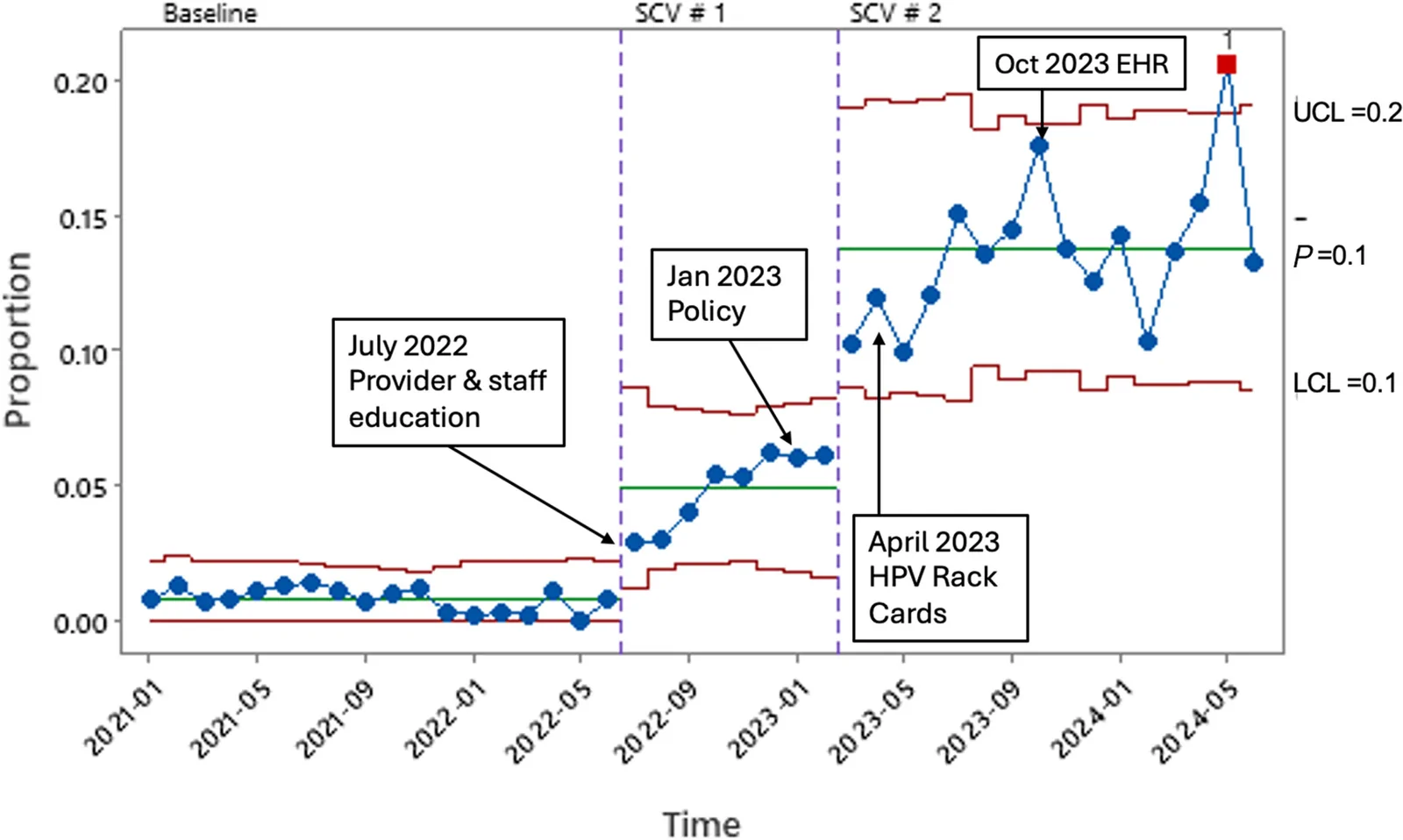
Statistical process control P-chart displaying the monthly proportion of eligible patients receiving the first dose of the HPV vaccine from January 2021 through June 2024. Blue circles represent the observed monthly vaccination proportion, the green centerline indicates the mean proportion for each phase, and the red lines represent the UCL and LCL. Control limits were adjusted for unequal monthly sample sizes. Vertical dashed purple lines denote the centerline shifts following detection of SCV. The QI initiatives are annotated in the text boxes. Red squares indicate observations meeting criteria for SCV (outside the UCL). Two centerline shifts were observed: the first in mid-2022 following provider and staff education, and the second in early 2023 following implementation of the departmental vaccination policy. Tests are performed with unequal sample sizes. LCL, lower control limit; SCV, special cause variation; UCL, upper control limit.

To evaluate trends over time, a statistical process control P-chart was constructed to display HPV vaccine uptake from January 2021 through June 2024 (Fig. 2). Control charts were generated using Minitab (version 22.1; Minitab LLC, State College, PA). The charts included standard Shewhart control limits and a baseline fixed centerline based on preintervention data. Four improvement initiatives were annotated on the chart to contextualize key implementation phases. Special cause variation was assessed using established statistical process control rules, using the rules established by the Western Electric Company.^16^

**Fig. 3.**
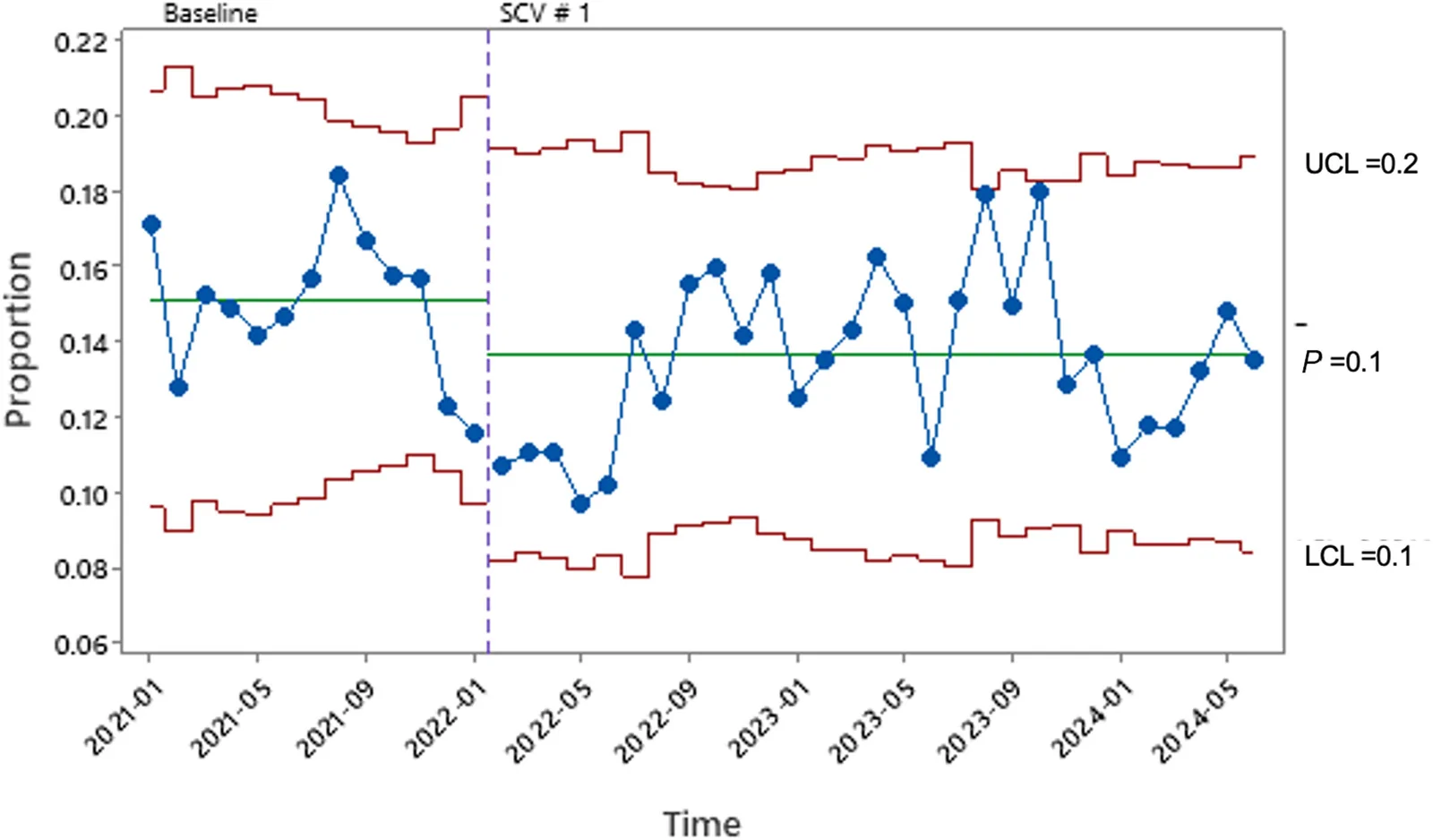
Statistical process Control P-chart of Tdap vaccine rates displaying the monthly proportion of eligible patients receiving the Tdap vaccine among patients aged 9–10 years from January 2021 through June 2024. Blue circles represent the observed monthly vaccination proportion, the centerline (green lines) indicates the mean proportion for each SCV, and the red lines represent the UCL and LCL. Control limits account for unequal monthly sample sizes. Vaccination rates remained largely within control limits, with 1 special cause signal detected in early 2022, before the start of any improvement initiative. Tests are performed with unequal sample sizes. LCL, lower control limit; SCV, special cause variation; UCL, upper control limit.

Finally, Tdap vaccination rates were assessed as a balancing measure using a parallel P-chart constructed with the same inclusion criteria and stratification (Fig. 3). This allowed for a comparison of temporal patterns in HPV and Tdap uptake to evaluate whether observed increases in HPV initiation reflected intervention-specific effects or a broader trend in early adolescent vaccination practices.

## RESULTS

The average monthly proportion of HPV first-dose vaccination increased from a baseline of roughly 2% to more than 14% following the second process shift, exceeding the initiative’s aim of a 10 percentage-point increase. Figure 2 displays a P-chart of monthly HPV vaccine initiation among 9- and 10-year-olds from January 2021 through June 2024, covering the duration of the QI initiative and extending into the subsequent year to assess for sustained changes. Rates remained consistently low during the baseline period, with a modest upward shift emerging in mid-2022 following improvement initiative 1, focused on provider and staff education. More pronounced gains were observed in early 2023 after adoption of the institutional “Start at 9” policy and EHR modifications. To detect special cause variation, we used Western Electric Rules and identified 2 shifts in the centerline mean due to 8 or more data points being on 1 side of the centerline (Fig. 2). This analysis identified 2 shifts in the mean, the first in mid-2022 and the second in early 2023, both aligning with key improvement initiatives (Fig. 2). The latter shift reflected a stable and sustained improvement in performance. Together, these charts demonstrate a high probability that observed improvements were temporally aligned with intervention activities and led to sustained system-level change. One instance of special cause variation was identified near the end of the study period (May 2024), likely reflecting the beginning of the seasonal increase in well-child visits during this month.

To contextualize these findings, the balancing measure, defined as the proportion of children in the same age group who received the Tdap vaccine, was monitored. Unlike HPV, Tdap is a single-dose vaccine recommended at ages 11–12 years but can be administered at age 10. Vaccination rates remained largely within control limits, with only 1 special cause signal detected in early 2022, before the start of any intervention. When controlling for preintervention special cause variation, no further changes in Tdap vaccination rates were seen (Fig 3).

A total of 9,315 patients aged 9–10 years received at least 1 well-child visit across 6 pediatric primary care sites from 2021 to 2024. Table 1 summarizes the demographic characteristics of the eligible population, stratified by sex, race/ethnicity, and insurance type. Table 2 presents HPV vaccination rates for the first dose only, stratified by these same demographic subgroups. Overall vaccination rates increased substantially during the 3.5-year period, with the largest gains occurring between 2022 and 2023. By 2024, initiation rates were highest among Hispanic/Latino (19.8%) and Medicaid/CHIP children (17.1%). In contrast, White non-Hispanic children (12.4%) and those with private insurance (12.3%) had comparatively lower rates. No major unintended consequences were reported during the implementation level.

**Table 1.** Demographic Characteristics of 9- to 10-year-olds Included in the Study, Stratified by Sex, Race/Ethnicity, and Insurance Type (2021–2024)

Subgroup	2021, n (%)	2022, n (%)	2023, n (%)	2024, n (%)
Insurance				
Medicaid/CHIP	807 (32.8)	918 (35.3)	966 (38.5)	666 (38.0)
Private + military	1398 (56.8)	1520 (58.4)	1375 (54.8)	919 (52.4)
Uninsured	146 (5.9)	62 (3.5)	87 (3.4)	80 (4.5)
Unknown	110 (4.5)	72 (2.8)	82 (3.3)	86 (4.9
Race/ethnicity				
Asian NH	398 (16.2)	422 (16.2)	450 (17.9)	248 (14.2)
Black NH	202 (8.2)	229 (8.8)	241 (9.6)	161 (9.2)
White NH	1364 (56.4)	1444 (55.5)	1298 (51.7)	947 (54.1)
Other race NH*	116 (4.7)	115 (4.4)	108 (4.3)	66 (3.8)
Hispanic/Latino	271 (11.0)	290 (11.2)	316 (12.6)	258 (14.7)
Unreported	87 (3.5)	101 (3.9)	97 (3.9)	71 (4.1)
Sex				
Female	1136 (48.3)	1190 (47.6)	1252 (49.9)	857 (48.9)
Male	1215 (51.7)	1310 (52.4)	1255 (50.0)	892 (50.9)
Total	2458	2599	2507	1751

Private and military insurance were combined into a single category due to their similar insurance structures and the small number of patients with military insurance. Data from 2021 to 2023 were collected annually, whereas 2024 data were collected from January 1, 2024, to June 30, 2024, to reflect the study period.

* Other race NH includes non-Hispanic individuals without recoded race, as well as those who identified as non-Hispanic American Indian/Alaskan Native.

NH, non-Hispanic; CHIP, Children’s Health Insurance Program.

**Table 2.** HPV First-dose Vaccination Rates by Subgroup, 2021–2024 (%)

Subgroup	2021, %	2022, %	2023, %	2024, %
Insurance				
Medicaid/CHIP	0.25	3.70	10.04	17.12
Private + military	0.29	2.17	9.75	12.30
Uninsured	0.00	3.30	16.09	10.00
Unknown	0.00	1.39	10.84	10.47
Race/ethnicity				
Asian NH	0.50	1.90	9.11	17.34
Black NH	0.50	4.37	12.45	11.18
White NH	0.22	2.15	9.24	12.04
Other race NH*	0.00	5.22	8.33	4.55
Hispanic/Latino	0.00	4.48	13.61	19.77
Unreported	0.00	2.97	11.34	21.12
Sex				
Female	0.40	2.48	10.86	14.59
Male	0.08	2.98	9.40	13.34

Data from 2021 to 2023 were collected annually, whereas 2024 data were collected from January 1, 2024, to June 30, 2024, to reflect the study period.

* Other race NH includes non-Hispanic individuals without recoded race, as well as those who identified as non-Hispanic American Indian/Alaskan Native.

NH, non-Hispanic; CHIP, Children’s Health Insurance Program.

## DISCUSSION

This iterative QI initiative was associated with a sustained improvement in early HPV vaccine initiation. The P-charts demonstrate patterned improvements that corresponded with key phases of implementation, suggesting that the intervention modified workflows to drive vaccine delivery. Furthermore, the identification of 2 process shifts—including a sustained shift following provider and staff education and implementation of the “Start at 9” policy—suggests that the initiative meaningfully influenced how vaccination opportunities were recognized and acted upon, consistent with QI efforts from a learning collaborative–based study.^17^ Moreover, the stability of Tdap vaccination after implementation of the interventions further suggests that the rise in HPV initiation reflects targeted effects rather than a general shift in early adolescent vaccination practices.

Our findings are consistent with previous studies demonstrating improved early HPV vaccination rates following structured QI interventions.^18–20^ Similar to prior initiatives, our project included education for providers (attending physicians, nurse practitioners, physician assistants, and residents) on recommending HPV vaccination beginning at age 9 years.^18^ In addition, we expanded these efforts by educating clerical and clinical staff, including licensed practical nurses and medical assistants, about our ongoing QI initiatives.^18^ We also incorporated patient education materials focused on HPV vaccination at age 9 years, which likely reinforced provider recommendations and improved parental understanding of the benefits of early vaccination.^19^

Although we enhanced the EHR by adding HPV vaccine orders to well-child visit order sets for ages 9 years and older, this intervention could have been strengthened by modifying the EHR vaccine forecasting system to prompt providers and residents to initiate HPV vaccination at age 9 years.^19,20^ Unfortunately, this functionality is not currently customizable within our institution’s EHR system.

The interventions used in this QI initiative are feasible to maintain and adaptable to other pediatric and primary care contexts because they can be embedded into routine clinical workflows. The success of this initiative highlights the practical impact of aligning policy, technology, and patient engagement within a coordinated QI framework to improve preventive care delivery.

It is important to recognize the variation in vaccination rates by demographic subgroup, and how this discord highlights the importance of applying an equity lens to QI initiatives. According to our 2023 yearly data, Hispanic and Black non-Hispanic children had the highest rates of first-dose initiation, whereas White non-Hispanic children had one of the lowest rates. One explanation may be that racially diverse communities, particularly those with a history of targeted public health outreach, are more receptive to clinic-based interventions. Conversely, lower uptake among White non-Hispanic children may reflect hesitancy, less engagement with outreach, or sociopolitical influences. Recent literature has highlighted the role of political ideology and misinformation in influencing vaccine acceptance.^21^ Specifically, conservative political ideology has been associated with lower awareness and uptake of the HPV vaccine, potentially due to values-based resistance to vaccines tied to sexual health or distrust in government-sponsored public health campaigns.^22^ These factors, while not measured in our study, underscore the complexity of vaccine behavior and the need for demographically and contextually responsive approaches to early HPV vaccine promotion.

Although our interventions were effective, additional evidence-based strategies could further increase HPV vaccination uptake at age 9 years. These include the implementation of standing orders and provider assessment and feedback. Establishing a formal standing order policy could authorize eligible patients to receive the HPV vaccine without requiring a separate examination or direct order from the attending provider, thereby reducing missed opportunities for vaccination and improving workflow efficiency.^23^ A standing order policy within this study could not be implemented because there were no registered nurses at our ambulatory sites to carry out these vaccination orders. The clinical staff consists of licensed practical nurses and medical assistants.

Provider assessment and feedback involves evaluation of providers’ performance in recommending and administering the HPV vaccine to the eligible patient population and providing individualized feedback on their vaccination rates. This approach promotes accountability, identifies opportunities for improvement, and has been shown to increase provider adherence to vaccination recommendations and improve HPV vaccine uptake.^23,24^ We were unable to implement this intervention because of data reporting limitations, as aggregated data were reported by ambulatory office site rather than by individual provider. In addition, our practice included 21 providers and more than 30 resident physicians, making individualized performance reporting difficult during the study period.

This study has several limitations. First, the use of Tdap vaccination rates among 9- to 10-year-olds as a balancing measure may limit generalizability outside New York State. Although many states administer Tdap at ages 11–12 years, New York State requires Tdap vaccination for sixth-grade entry, and because school eligibility extends through December birthdays, some children receive Tdap vaccine at age 10 years. Consequently, early HPV initiation and Tdap receipt overlapped within our cohort. Second, as a QI initiative conducted within a single health system, our findings may not be generalizable to other settings with different patient populations, resource constraints, or organizational structures. Finally, although the dataset included key demographic variables, other important factors, such as parental attitudes, strength of provider recommendation, and clinic-level operational changes, were not captured and may have influenced vaccine uptake in ways not reflected in the analysis.

Despite these limitations, this initiative offers several strengths. Our analysis introduced the use of an internal benchmark not previously described in the literature by using Tdap vaccination as a comparator vaccine. Additionally, although our interventions were similar to those used in prior studies, we were the first to implement system-level changes through the formal adoption of the “Start at 9” policy.^17–20^ Furthermore, unlike earlier work that examined broader age ranges, our study focused specifically on patients aged 9 and 10 years. This enabled us to identify targeted demographic differences in initiation patterns, including that White patients in our suburban cohort were less likely to begin vaccination at these ages. Such observations add nuance to existing evidence and offer hypotheses regarding the influence of local context and community beliefs on vaccine uptake.

Future work should use more rigorous analyses to better characterize subgroup differences and extend follow-up to assess series completion. Strategies to incorporate measures of parental attitudes, provider communication, and site-level variation should also be considered. These steps will improve causal inference, help guide targeted interventions, and inform the design of equity-focused strategies to advance HPV vaccine uptake and cancer prevention.

## CONCLUSIONS

This study demonstrates the effectiveness of a systems-level QI approach that integrated evidence-based strategies through a coordinated, multidisciplinary effort. Interventions included institutional policy change, provider education, patient-facing educational initiatives, and EHR modifications. As part of the “Start at 9” campaign, these activities were associated with a measurable and sustained increase in HPV vaccine initiation among 9- and 10-year-olds, supporting early vaccination as a feasible and effective practice.

## References

[1] Patel C Brotherton JM Pillsbury A. The impact of 10 years of human papillomavirus (HPV) vaccination in Australia: what additional disease burden will a nonavalent vaccine prevent? Euro Surveill. 2018;23:1700737. 30326995 10.2807/1560-7917.ES.2018.23.41.1700737 PMC6194907

[2] Falcaro M Castañon A Ndlela B. The effects of the national HPV vaccination programme in England, UK, on cervical cancer and grade 3 cervical intraepithelial neoplasia incidence: a register-based observational study. Lancet. 2021;398:2084–2092. 34741816 10.1016/S0140-6736(21)02178-4

[3] Lei J Ploner A Elfström KM. HPV vaccination and the risk of invasive cervical cancer. N Engl J Med. 2020;383:1340–1348. 32997908 10.1056/NEJMoa1917338

[4] Nielsen KJ Jakobsen KK Jensen JS. The effect of prophylactic HPV vaccines on oral and oropharyngeal HPV infection-a systematic review. Viruses. 2021;13:1339. 34372545 10.3390/v13071339 PMC8310210

[5] Macilwraith P Malsem E Dushyanthen S. The effectiveness of HPV vaccination on the incidence of oropharyngeal cancers in men: a review. Infect Agent Cancer. 2023;18:24. 37095546 10.1186/s13027-022-00479-3 PMC10127083

[6] Perkins RB Humiston S Oliver K. Evidence supporting the initiation of HPV vaccination starting at age 9: collection overview. Hum Vaccin Immunother. 2023;19:2269026. 37824444 10.1080/21645515.2023.2269026 PMC10572037

[7] Bednarczyk RA Brandt HM. Descriptive epidemiology of age at HPV vaccination: analysis using the 2020 NIS-Teen. Hum Vaccin Immunother. 2023;19:2204784. 37138466 10.1080/21645515.2023.2204784 PMC10161935

[8] O’Leary ST. Why the American Academy of Pediatrics recommends initiating HPV vaccine at age 9. Hum Vaccin Immunother. 2022;18:2146434. 36404635 10.1080/21645515.2022.2146434 PMC9746363

[9] American Cancer Society updates HPV vaccination guideline, making adaptations from 2019 federal advisory committee. American Cancer Society MediaRoom. 2020. Available at https://pressroom.cancer.org/HPVguideline2020. Accessed October 12, 2025.

[10] New York State Department of Health. New York State Prevention Agenda 2025–2030 State Dashboard. 2025. Available at https://apps.health.ny.gov/public/tabvis/PHIG_Public/pa/reports/#state. Accessed December 14, 2025.

[11] Nguyen KH Santibanez TA Stokley S. Parental vaccine hesitancy and its association with adolescent HPV vaccination. Vaccine. 2021;39:2416–2423. 33775438 10.1016/j.vaccine.2021.03.048 PMC8066400

[12] Bednarczyk RA Brewer NT Gilkey MB. Human papillomavirus vaccination at the first opportunity: an overview. Hum Vaccin Immunother. 2023;19:2213603. 37218520 10.1080/21645515.2023.2213603 PMC10294726

[13] Zorn S Darville - Sanders G Vu T. Multi-level quality improvement strategies to optimize HPV vaccination starting at the 9-year well child visit: success stories from two private pediatric clinics. Hum Vaccin Immunother. 2023;19:2163807. 36798976 10.1080/21645515.2022.2163807 PMC10054168

[14] Brandt HM Footman A Adsul P. Implementing interventions to start HPV vaccination at age 9: using the evidence we have. Hum Vaccin Immunother. 2023;19:2180250. 36803261 10.1080/21645515.2023.2180250 PMC10026886

[15] New York State HPV Coalition. HPV vaccination is cancer prevention. New York State HPV Coalition. 2018. Available at https://www.nyshpv.org/. Accessed October 12, 2025.

[16] Western Electric Company. Statistical Quality Control Handbook. Western Electric Co.; 1956.

[17] Oliver K Beskin K Noonan L. A quality improvement learning collaborative for human papillomavirus vaccination. Pediatr Qual Saf. 2021;6:e377. 33409429 10.1097/pq9.0000000000000377 PMC7781351

[18] Brodie N McPeak KE. Improving human papilloma virus vaccination rates at an urban pediatric primary care center. Pediatr Qual Saf. 2018;3:e098. 30584625 10.1097/pq9.0000000000000098 PMC6221589

[19] Bowden M Yaun J Bagga B. Improving human papilloma virus vaccination rates: quality improvement. Pediatr Qual Saf. 2017;2:e048. 30229184 10.1097/pq9.0000000000000048 PMC6132894

[20] Goleman MJ Dolce M Morack J. Quality improvement initiative to improve human papillomavirus vaccine initiation at 9 years of age. Acad Pediatr. 2018;18:769–775. 29842924 10.1016/j.acap.2018.05.005

[21] Lim J Moon KK. Political ideology and trust in government to ensure vaccine safety: using a U.S. survey to explore the role of political trust. Int J Environ Res Public Health. 2023;20:4459. 36901469 10.3390/ijerph20054459 PMC10002444

[22] Chido-Amajuoyi OG Onyeaka H Amonoo HL. The influence of political ideology on awareness of HPV and HPV vaccine among adults in the United States. Hum Vaccin Immunother. 2023;19:2232706. 37529922 10.1080/21645515.2023.2232706 PMC10399468

[23] Isher-Witt J Foley S Hassan A. Age nine is possible: improving age 9 HPV initiation through a national quality improvement initiative during the COVID-19 pandemic. Hum Vaccin Immunother. 2023;19:2284359. 37994120 10.1080/21645515.2023.2284359 PMC10760390

[24] Perkins RB Legler A Jansen E. Improving HPV vaccination rates: a stepped-wedge randomized trial. Pediatrics. 2020;146:e20192737. 32540986 10.1542/peds.2019-2737

